# Evaluation of a Haptic Virtual Reality Simulator and Its Impact on Preclinical Dental Education

**DOI:** 10.7759/cureus.109623

**Published:** 2026-05-25

**Authors:** Sandra Erika Ibarra-Martínez, Miguel Ángel Noyola-Frías, Francisco Javier Aguilar-Zapata, Carolina Rivera-Luque, Ricardo Martínez-Rider, José Manuel Alderete-Hernández, Selene Velázquez-Moreno, Marlen Vitales-Noyola

**Affiliations:** 1 Haptic Simulation Laboratory, Faculty of Dentistry, Autonomous University of San Luis Potosi, San Luis Potosí, MEX; 2 Oral and Maxillofacial Surgery, Faculty of Dentistry, Autonomous University of San Luis Potosi, San Luis Potosí, MEX; 3 Department of Health and Sport Sciences, TUM School of Medicine and Health, Technical University of Munich, Munich, DEU; 4 Pharmaceutical-Biological Sciences, Faculty of Chemical Sciences, Autonomous University of San Luis Potosí, San Luis Potosí, MEX; 5 Endodontics, Faculty of Dentistry, Autonomous University of San Luis Potosi, San Luis Potosí, MEX

**Keywords:** dental students, haptic, perception, preclinic training, simodont

## Abstract

Background: Haptic simulation is increasingly being integrated into health education due to its ability to provide a realistic tactile experience, enhancing skill acquisition, student confidence in clinical settings, and reducing iatrogenic risks. In dentistry, this tool complements traditional teaching methodologies by facilitating hands-on preclinical training. This study aimed to evaluate students' perceptions regarding the use of haptic simulation in their learning process and preclinical skill development.

Methods: A scientifically validated 10-question survey was administered to students at the Autonomous University of San Luis Potosí who had undergone training with dental phantoms, followed by practice with the Simodont® haptic simulator (Nissin Dental Products BV, Amsterdam, the Netherlands). The survey was conducted via an online platform (Google Forms, Google LLC, CA, USA), yielding responses from 125 students from the fifth semester onwards.

Results: The results indicate that 75% (n = 94) of the students perceive a significant improvement in their manual skills and abilities, 79.5% (n = 99) consider haptic simulation an effective tool for self-directed learning, and 79.6% (n = 100) report increased confidence in performing cavity preparations on real patients after training with Simodont®.

Conclusions: These findings suggest that haptic simulation serves as a valuable adjunct to conventional preclinical training in dental education, reinforcing both technical proficiency and student confidence before clinical practice.

## Introduction

Dentistry is a healthcare field in which professionals face multiple challenges in their daily clinical practice. Dentists must perform invasive procedures in very small, poorly lit working areas that are potentially contaminated with microorganisms capable of causing infections. In addition, these procedures require the use of sharp instruments operating at high speeds, such as handpieces and scalpels, making the job even more complex [1]. To ensure meaningful learning and protect patients, dental schools have implemented preclinical training methods that recreate real-life scenarios. These methods involve the use of simulators installed in environments that closely resemble clinical settings, providing students with a realistic and safe training experience [1].

Teaching dental students requires integrating clinical practice into the classroom as an effective learning strategy that bridges the gap between theory and practice. Therefore, dental schools must establish preclinical laboratories for hands-on training with simulators. Initially, teaching methods relied on students working with inert objects, such as plastic models that replicated human jaws [2]. These models were later placed into mannequins that mimicked patient positions and featured jointed, adjustable movements for mouth opening, allowing modifications to the head, neck, and torso positions. This setup enabled students to develop ergonomic skills, adjust patient positioning, and practice various dental treatments using models mounted on accessible dental arches (typodonts) that simulated an open mouth [2]. Over time, with the advancement of new technologies, the need arose to seek better alternatives in dental education, beyond synthetic models that bore little resemblance to real oral structures [3]. The phantom head has become the most widely used and established method in dental schools, offering multiple advantages and serving as a simple yet effective teaching tool [4]. The significant technological advancements of recent years, particularly the early exposure to technology in education, have highlighted the necessity of supporting the learning of fundamental skills in dentistry with modern resources and more advanced simulators [5-7].

Medicine was one of the first fields to benefit from the introduction of haptic technology through various touch-based devices with clinical diagnostic applications. These devices enable the reproduction of surgical sensitivity with high realism, offering greater safety for patient use and reducing environmental impact, as they generate little to no plastic waste [4,8-10]. In dentistry, several technological advancements have also been introduced to improve patient treatment. One such development is haptic simulation, which allows users to feel and manipulate tools and organs in a low-risk virtual environment. It also enables the performance of various procedures, such as tissue cutting, with high tactile realism [4,11]. Furthermore, haptic simulation technology has been adapted for preclinical training in dental schools, enhancing students' hands-on learning experiences. Traditional preclinical dental education relies heavily on the use of artificial and consumable materials, which may generate considerable economic costs and environmental waste. Artificial teeth and plastic models often require replacement after procedural errors, limiting opportunities for repeated practice and increasing material consumption. In this context, haptic virtual reality simulators provide a sustainable and cost-effective alternative by enabling unlimited procedural repetition in a controlled and safe educational environment without the continuous use of disposable materials.

In this regard, the Moog Industrial Group of Amsterdam developed the dental homework trainer known as Simodont® (Nissin Dental Products BV, Amsterdam, the Netherlands), making it one of the first haptic simulators in dentistry [4]. Simodont® is a recently introduced device that differs from traditional task simulators and offers multiple advantages for dental education. It allows for the reproduction of pathologies and provides enhanced magnification capabilities [4,12]. In addition, Simodont® offers further benefits through its integration with an intelligent operating system. This system enables the evaluation of student performance via a standardized measurement process that assesses drilling in both permitted and restricted wear areas. It also captures and stores images of tooth preparations, arch work within the virtual cube, or cylindrical virtual laboratory models at different time points. This feature allows educators to monitor students' progress in preclinical training [13]. Since its introduction into dentistry and its development in the Netherlands, Simodont® has become an integral part of dental schools worldwide. It is now used in institutions across the United States, Australia, and various European countries such as Spain, Germany, and the United Kingdom. Additionally, it has been adopted in several Asian nations, including Saudi Arabia, China, and Turkey, as well as in Latin American countries such as Brazil and Chile. Recently, Simodont® has also been introduced in Mexico. In Mexico, this haptic device for preclinical training was recently introduced at the Faculty of Dentistry in San Luis Potosí. Two years ago, the haptic simulation laboratory was inaugurated, providing both undergraduate and postgraduate dental students with the opportunity to practice various treatments, including cariology, prosthodontics, and endodontic access, among others, using this advanced simulator.

The Simodont® incorporates AI-based components primarily through adaptive algorithms and data-driven feedback systems that analyze user performance in real time [4] and record parameters, such as handpiece movement, applied force, preparation depth, and spatial accuracy, comparing them with predefined ideal models derived from expert procedures. Based on this analysis, the system automatically generates objective performance scores and personalized feedback, enabling self-directed learning [4]. In addition, the virtual environment employs intelligent collision detection and tissue response modeling to simulate realistic cutting behavior of dental tissues. Although not a fully autonomous AI system, these adaptive and analytics-driven features allow Simodont® to function as an intelligent training platform that supports individualized skill acquisition and competency-based dental education.

This study aimed to assess dental students’ perceptions of Simodont in preclinical dental training, especially its usefulness for manual skill development, self-learning, and confidence in cavity preparation.

## Materials and methods

Participants and ethical considerations

This study was conducted in accordance with ethical guidelines. It was approved by the Ethics and Research Committee of the Faculty of Dentistry at the Autonomous University of San Luis Potosí, under the following protocol code: CEI-FE-03-024. All participants were thoroughly informed about the purpose, procedures, and benefits of the study before agreeing to take part. Participation was entirely voluntary, and students had the right to withdraw at any stage without any academic or personal consequences. A non-probabilistic sampling method was used, and a total of 125 participants were included in the study. Each participant provided written informed consent before completing the questionnaire. To ensure confidentiality and compliance with data protection regulations, all responses were anonymized, and no personally identifiable information was collected. The data was securely stored and used exclusively for research purposes. This observational study adheres to the principles outlined in the Declaration of Helsinki for ethical research involving human participants.

Simodont dental simulator

The Simodont® Dental Trainer (Nissin Dental Products BV, Amsterdam, the Netherlands) is a haptic virtual reality simulator designed for preclinical dental education. The system integrates a feedback haptic device, stereoscopic 3D visualization, and dedicated simulation software that allows students to perform virtual dental procedures under realistic tactile and visual conditions. The equipment consists of a computerized workstation equipped with dual stereoscopic screens, a phantom head interface, and a haptic handpiece that replicates the sensation of dental instruments interacting with hard and soft tissues. The system provides real-time visual and haptic feedback, enabling the simulation of a wide range of dental procedures, including cavity preparation, caries removal, access cavity design, as well as selected periodontal and implantology procedures. The Simodont® software includes an interactive learning platform that allows instructors to assign specific clinical scenarios, monitor student performance, and provide structured feedback. Performance metrics such as accuracy of preparation, removal of tooth structure, and procedural time can be recorded by the system, allowing objective tracking of student progress in preclinical training. In the present study, Simodont® was used as a complementary educational tool for preclinical training in restorative dentistry, supporting the development of psychomotor skills in a controlled and standardized virtual environment prior to clinical patient exposure. The haptic simulation laboratory is equipped with two Simodont® units, corresponding to the 2021 and 2024 models, respectively, which are used for student training and skill development in a controlled virtual environment. In the present study, the Simodont® system was used as a complementary educational tool in preclinical dental training, supporting the development of psychomotor skills prior to clinical exposure to patients.

Survey preparation

A 10-question survey was developed based on previously validated questionnaires from scientific literature. The survey instrument used in this study was developed based on previous literature related to haptic simulation, virtual reality technologies, and educational perception in dental training environments. The questionnaire items were designed to evaluate students’ perceptions regarding usability, realism, educational usefulness, psychomotor skill development, and overall satisfaction with the Simodont® haptic simulator. The instrument consisted of structured, closed-ended questions using a Likert-type scoring system adapted to assess the degree of agreement and perceived educational value of the simulation experience. Prior to its implementation, the questionnaire was reviewed by faculty members with expertise in dental education and simulation-based training to assess content relevance, clarity, and coherence of the items. Minor modifications were performed according to their recommendations to improve comprehensibility and applicability, according to the educational context, objectives of the study, and language requirements of our student population to ensure clarity, relevance, and comprehensibility for the participants. The scoring system was designed to identify levels of perceived usefulness and acceptance of the technology within the preclinical educational environment. Reliability and internal consistency considerations were included during the survey design process to support the methodological rigor of the instrument. The survey was conducted using Google Forms (Google LLC, CA, USA) and distributed to students via institutional email and WhatsApp. The participants who completed the survey were fifth-semester students and beyond, meaning they had completed at least one full semester of preclinical training with Simodont® (Nissin Dental Products BV). To ensure effective distribution and participation, the faculty's academic authorities, through the academic secretary, facilitated the process by informing and inviting students to respond to the survey. A total of 125 students participated in the study (questionnaire was added in the supplemental information; see Appendix). 

Data collection and management

All data obtained from the questionnaires were systematically recorded in a Microsoft Excel database (Microsoft Corp., USA) to facilitate efficient data management, organization, and analysis. The database was structured to ensure accuracy, allowing for the classification and interpretation of responses. The collected data serve as the foundation for statistical evaluation, enabling a comprehensive assessment of students' perceptions regarding the use of Simodont® in their preclinical training. 

Statistical analysis

Statistical analysis was performed using GraphPad Prism v5.0 software (GraphPad Software, San Diego, CA). All variables were previously coded and classified according to their nature as continuous or ordinal. In the case of questionnaire-based variables (Likert-type scale responses), data were treated as ordinal variables and analyzed accordingly. Prior to statistical comparisons, normality of continuous variables was assessed using the Shapiro-Wilk test. Categorical variables were summarized as frequencies and percentages. For Likert-scale items, central tendency measures were reported as median and interquartile range (Q1-Q3), given their ordinal distribution. When appropriate, composite scores derived from grouped items (e.g., perception domains such as usefulness, satisfaction, or perceived skill improvement) were treated as continuous variables after verification of internal consistency. For comparisons between two independent groups, Student’s t-test was applied for normally distributed continuous variables, while the Mann-Whitney U test was used for ordinal or non-parametric data. In cases involving paired or pre- and post-intervention evaluations (when applicable in the study design), a paired Student’s t-test or Wilcoxon signed-rank test was used depending on data distribution. For comparisons among three or more groups (e.g., different academic levels or exposure categories to Simodont), one-way ANOVA with Tukey’s post hoc test was used for parametric data, whereas the Kruskal-Wallis test followed by Dunn’s multiple comparison test was applied for non-parametric variables. All tests were two-tailed, and a p-value <0.05 was considered statistically significant.

## Results

Students' self-perception of theoretical knowledge and ergonomics in haptic simulation

**Figure 1:**
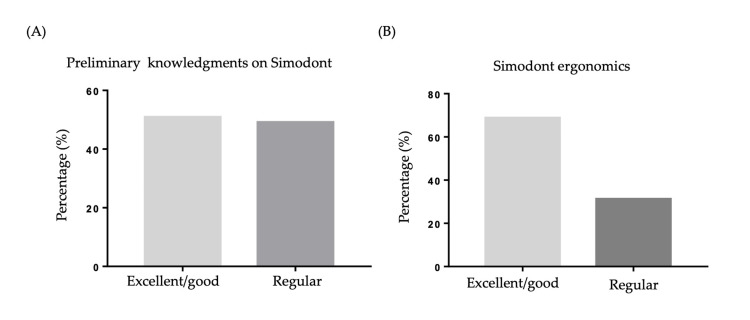
Ergonomics and theoretical knowledge before practice. The initial survey assesses students' theoretical knowledge and perceptions before using the Simodont simulator. (A) Percentage of students who mention that theoretical knowledge on the use of Simodont is necessary before starting to use it. (B) Percentage of students who assess the ergonomic suitability, as excellent/good and regular. Error bars represent the mean ± standard deviation (n = 125).

The initial section of the survey aimed to assess students' self-perception regarding their theoretical knowledge of Simodont® and to evaluate the ergonomic suitability of the device for cavity preparation. Results indicated that 50.9% (n = 63) of students rated their theoretical knowledge as excellent/good, considering it beneficial for adapting to Simodont®, whereas 49.1% (n = 62) did not perceive theoretical knowledge as a critical factor; no statistically significant difference was observed in this aspect (p > 0.05). Regarding ergonomics, 68.75% (n = 86) of the students considered Simodont® to be ergonomically suitable for cavity preparation, while 31.25% (n = 39) found as regular. In this case, a statistically significant difference was observed (p < 0.05) (Figure 1A, 1B).

Students' perception of Simodont®, manual skill acquisition, and clinical confidence

**Figure 2:**
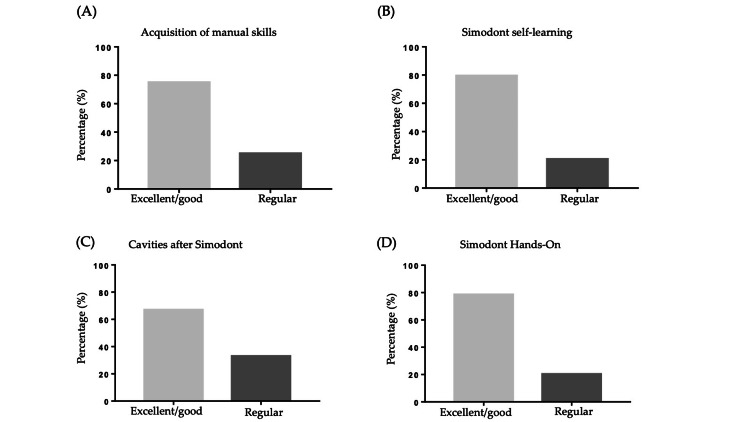
Acquisition of manual skills and self-learning. This figure presents survey responses related to the acquisition of manual skills and self-learning. (A) Percentage of students who consider Simodont useful for acquiring manual skills, as excellent/good and regular. (B) Percentage of students who find Simodont training beneficial for self-directed learning, as excellent/good and regular. (C) Percentage of students who feel they have the necessary skills to perform cavity preparations in clinical practice after using Simodont for preclinical training, as excellent/good and regular. (D) Percentage of students reflecting on their overall experience performing cavity preparations, as excellent/good and regular. Error bars represent the mean ± standard deviation (n = 125).

The following section of the survey aimed to evaluate the effectiveness of Simodont® training in manual skill acquisition, self-learning, clinical cavity preparation, and overall student experience. The results revealed that 75% (n = 94) of the students considered Simodont® an excellent/good tool for developing manual skills, while 25% (n = 31) rated it as moderate (p < 0.05). Meanwhile, 79.5% (n = 99) of the students perceived Simodont® as excellent/good for promoting self-learning, whereas 20.5% (n=26) did not report significant improvements (p < 0.05). Sixty-seven percent (67%; n = 84) of the students felt confident performing cavity preparations in a clinical setting after training with Simodont®, while 33% (n = 41) rated their confidence as moderate (p < 0.05). Moreover, 79.6% (n = 100) of the students described their overall experience with cavity preparation as excellent/good, while 21.4% (n = 25) did not find the experience significantly beneficial (p < 0.05) (Figure 2).

Haptic feedback in Simodont®

A Comparison With Typodonts and Real Teeth

**Figure 3:**
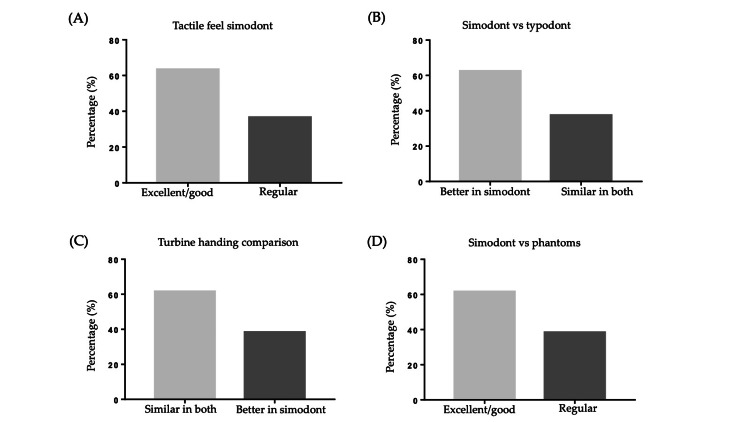
Comparison of overall perception and handpiece experience with Simodont vs. traditional methods. This figure presents survey responses regarding students' perception of using the Simodont simulator. (A) Percentage of students who evaluate how similar the sensation of using the Simodont handpiece is to working on a real tooth. (B) Percentage of students who assess how closely the sensation of the Simodont handpiece resembles that of working on a typodont tooth, as excellent/good and regular. (C) Percentage of students who compare the resemblance of the Simodont turbine to a dental unit turbine. (D) Percentage of students who perceive working with the Simodont as more realistic compared to phantom models. Error bars represent the mean ± standard deviation (n = 125).

The final section of the survey aimed to evaluate the perceived similarity of the Simodont® handpiece and turbine in comparison to traditional dental training methods, including real teeth and typodonts. In addition, students' perceptions of Simodont® were compared to conventional phantom models. The results indicated that 62.5% (n = 78) of the students rated the similarity between the Simodont® handpiece and a typodont tooth as excellent/good, while 38.4% (n = 47) considered it moderate (p < 0.05). Meanwhile, 63.4% (n = 79) of students found the similarity between the Simodont® handpiece and a real tooth to be excellent/good, whereas 36.6% (46) rated it as moderate (p < 0.05). In addition, 61.6% (n = 77) of students rated the Simodont® turbine sensation as excellent/good in terms of similarity to a real turbine, while 38.4% (n = 48) did not perceive a significant resemblance (p < 0.05). Finally, 61.6% (n = 77) of students considered Simodont® superior to conventional phantom models, while 38.4% (n = 48) did not perceive a significant difference (p < 0.05) (Figure 3).

## Discussion

The integration of technology in dental education has revolutionized the way students acquire essential clinical skills. Simulation and virtual reality have emerged as powerful tools in teaching, learning, and clinical professional training, enabling students to develop and improve their abilities in a controlled, risk-free environment [14]. A fundamental aspect of dental education is the acquisition of psychomotor skills, as precision and hand-eye coordination are crucial for performing clinical procedures. To enhance these skills, the Simodont® dental trainer offers an innovative platform where students can practice manual dexterity with high fidelity and unlimited time. By allowing repeated practice before engaging in conventional clinical simulations, this technology helps students build confidence and security, ultimately improving their readiness for real-world patient care [14].

Our results indicate that, due to the highly dynamic nature of the Simodont® platform, extensive prior knowledge is not necessary for its effective use, as it is intuitive and easy to handle. This feature makes it particularly accessible to students at different levels of training, allowing them to familiarize themselves with the system quickly and focus on skill development rather than struggling with complex initial instructions. In addition, it is important to highlight that modern students interact with technology on a daily basis and are highly accustomed to using digital tools [15,16]. Their familiarity with virtual environments, simulations, and interactive interfaces significantly facilitates their adaptation to Simodont®, making the learning process smoother and more engaging. This technological affinity also enhances their motivation and confidence in using the system for training, as they perceive it as a natural extension of the digital tools they already use in other aspects of their education [16-18]. Regarding its ergonomic design and user comfort, the majority of students (almost 70%) report that Simodont® provides a proper fit and is a well-designed, user-friendly device. Its structure allows for extended periods of practice without causing discomfort or fatigue, making it an efficient tool for developing the necessary dexterity and precision in dental procedures. The ability to repeatedly practice techniques in a comfortable and controlled environment is a crucial factor in reinforcing manual skills, ultimately improving their clinical performance and preparedness for real-world scenarios [18,19]. Although students reported positive perceptions regarding the use of Simodont, the findings should be interpreted cautiously. The present study evaluated subjective opinions and self-reported experiences rather than objective measurements of psychomotor performance or clinical competence. Therefore, the perceived advantages of the simulator may not necessarily reflect actual improvements in clinical skills or academic outcomes. In addition, the questionnaire design and the predominance of favorable response categories may have contributed to positive response bias. These limitations highlight the importance of complementing students’ perceptions with objective performance assessments, control groups, and longitudinal evaluations in future studies to better determine the educational effectiveness and clinical relevance of haptic simulation technologies. 

Regarding the acquisition of manual skills through the use of Simodont®, the majority of students (almost 80%) state that it is a valuable tool for developing manual dexterity, as it allows them to practice as many times as necessary. Additionally, the software provides numerical feedback, enabling students to identify specific areas where they make mistakes and correct them accordingly [20]. In this regard, most students also mention that Simodont® is an excellent or good tool for self-learning (80%), as it facilitates independent skill improvement and continuous progress monitoring.

Several authors have evaluated the execution of this type of procedure, as well as others, by comparing cavity preparations performed using this equipment with those performed on typodonts or real teeth [21-24]. Across all studies, there is a consensus that cavity preparation using the simulator is superior to traditional methods. One of the key advantages of the software is its ability to highlight the areas to be worked on using color indicators, as well as provide real-time feedback on errors, working time, and performance. Since students can repeat the procedure an unlimited number of times, they have the opportunity to refine their technique until they master it. This feature represents a significant advantage, as it allows students to practice extensively and gain confidence before working with actual patients [25].

Regarding the use of the handpiece, students mentioned that the experience in the Simodont® is quite similar to real clinical practice. This is one of the main advantages of haptic simulation, as it allows virtual reality to replicate the use of dental instruments with realistic tactile feedback [26]. Additionally, students reported that they prefer using the Simodont® over typodonts, since the haptic simulator provides a more realistic sensation, closer to that of working on natural teeth. In this regard, several studies have compared both preclinical training methods, showing very favorable results for the Simodont® [27-30]. However, it is important to note that the use of haptic simulation continues to be considered a complementary tool within dental education curricula in many countries.

The graphical representation used in this study was intentionally selected to prioritize simplicity, readability, and rapid interpretation of the overall response trends among participants. Considering that the primary objective of the study was to evaluate general perceptions and acceptance of the haptic virtual reality simulator within the educational environment, simplified graphical formats were considered more appropriate for clearly communicating the predominant response patterns. Although stacked bar charts may provide a more detailed visualization of Likert-scale distributions, their use could increase visual complexity and reduce interpretability for readers when presenting multiple survey items simultaneously. Nevertheless, complete numerical distributions and percentages for each response category were included in the manuscript to ensure transparency and comprehensive interpretation of the collected data. In addition, the questionnaire design may have introduced a positive response bias, as the response options were limited to favorable categories (excellent, good, and fair) without including neutral or negative alternatives. This limitation may have influenced participants toward more positive evaluations and reduced the variability of responses. Therefore, the findings should be interpreted cautiously, as they primarily reflect students’ subjective perceptions and satisfaction levels rather than a fully balanced assessment of the Simodont experience. Future studies should consider incorporating a broader Likert scale with neutral and negative response options to improve the validity and interpretability of the results. Likert-type questionnaire responses are ordinal in nature, and therefore, the use of mean ± standard deviation may present methodological limitations in interpreting participants’ perceptions. Although these measures are frequently reported in educational research to summarize trends in responses, the results should be interpreted with caution. For this reason, the present study emphasizes descriptive analyses based on frequencies and percentages to provide a clearer representation of students’ perceptions.

Despite the educational advantages associated with haptic virtual reality simulation, several barriers related to the implementation of Simodont® systems in dental education have been reported in the literature. Among the most relevant challenges are the high acquisition and maintenance costs, infrastructure requirements, software updates, and the need for continuous faculty training to ensure effective integration into preclinical curricula. Additionally, some students may initially experience difficulties adapting to virtual environments and haptic feedback systems, particularly when transitioning from conventional phantom-head training models. Previous studies have emphasized that, although haptic simulation technologies improve repetitive practice opportunities and psychomotor skill development, they should be considered complementary educational tools rather than complete replacements for traditional preclinical training methods. According to Serrano CM et al. in “Virtual Reality and Haptics in Dental Education: Implementation Progress and Lessons Learned After a Decade” [20], the long-term incorporation of these technologies requires curricular adaptation, institutional investment, and continuous evaluation of educational outcomes to maximize their effectiveness in dental education. In the context of our institution and country, these challenges are particularly relevant, as access to advanced simulation technologies remains limited in public dental schools in Mexico. Nevertheless, the implementation of Simodont® has represented an important step toward modernization and innovation in preclinical dental education within our regional academic setting [20].

This study has several limitations that should be considered when interpreting the results. Since the sampling was derived from a single institution, this may limit the generalizability of the findings to other educational settings. The study design relied primarily on self-reported data, which may be subject to response bias and individual perception. Additionally, the absence of a control group and the short-term evaluation period preclude definitive conclusions regarding long-term skill acquisition and transfer to clinical performance. Future studies with larger, multicenter samples, longitudinal follow-up, and objective clinical outcome measures are recommended to further validate these findings. In addition, this study is related to the survey response scale used to evaluate students’ perceptions of the haptic virtual reality simulator. The instrument was specifically designed to assess levels of acceptance, perceived usefulness, and educational value among participants exposed to the simulation experience; therefore, the scale focused primarily on positive-performance evaluation ranges. Similar Likert-type approaches have been reported in studies assessing satisfaction and usability of simulation technologies in dental education. However, the restricted response range may have limited the identification of neutral or unfavorable perceptions. Future studies should consider broader evaluation scales to obtain a more comprehensive assessment of student experiences and perceptions regarding haptic simulation systems.

## Conclusions

The use of simulation-based training in dental education is perceived by students as a valuable tool for enhancing preclinical instruction. The Simodont® dental trainer provides a controlled and interactive environment that allows students to practice and develop manual dexterity through repeated simulation exercises. Based on self-reported questionnaire data, students expressed positive perceptions regarding its usefulness for skill practice, precision development, and confidence building prior to traditional preclinical training. However, given the descriptive design of the study, the absence of a control group, and the lack of objective performance measures, these findings reflect students’ perceptions rather than demonstrated improvements in clinical skills or patient care readiness. Further studies using objective assessments and comparative designs are required to evaluate the actual educational and clinical impact of this technology.

## Appendices

Survey to evaluate the impact of haptic simulation as a teaching method in dental education

Please carefully answer the following questions. Mark your preferred option with an X, using a scale from 8 to 10, where:

10 = Excellent / Very high 9 = Good 8 = Fair / Moderate

1. Before the practical training with Simodont, did you consider yourself theoretically prepared to use the simulator?

Excellent 10 Good 9 Fair 8

Justify your answer:

2. To what extent was the Simodont training useful for the development of your manual skills?

Excellent 10 Good 9 Fair 8

Justify your answer:

3. To what extent was the Simodont training useful for your self-directed learning?

Excellent 10 Good 9 Fair 8

Justify your answer:

4. To what extent does the sensation of using the Simodont handpiece resemble the use of a handpiece on a real tooth?

Excellent 10 Good 9 Fair 8

Justify your answer:

5. To what extent does the sensation of using the Simodont handpiece resemble the use of a handpiece on a typodont tooth?

Excellent 10 Good 9 Fair 8

Justify your answer:

6. To what extent does the Simodont turbine resemble the turbine of a dental unit?

Excellent 10 Good 9 Fair 8

Justify your answer:

7. How comfortable did you feel using Simodont to perform cavity preparations?

Excellent 10 Good 9 Fair 8

Justify your answer:

8. After using Simodont as preclinical training for cavity preparation, do you consider that you have the necessary foundations to perform cavity preparations in the clinic?

Excellent 10 Good 9 Fair 8

Justify your answer:

9. After working with Simodont, do you consider that the experience provides better perception compared with phantom simulators?

Excellent 10 Good 9 Fair 8

Justify your answer:

10. Overall, how would you rate your experience using Simodont for cavity preparation?

Excellent 10 Good 9 Fair 8

Justify your answer:
